# Progress Toward Poliomyelitis Eradication — Afghanistan and Pakistan, January 2013–August 2014

**Published:** 2014-10-31

**Authors:** Noha H. Farag, James Alexander, Stephen Hadler, Arshad Quddus, Elias Durry, Mufty Zubair Wadood, Rudolph H. Tangermann, Derek Ehrhardt

**Affiliations:** 1 Global Immunization Division, Center for Global Health, CDC; 2 Division of Viral Diseases, National Center for Immunization and Respiratory Diseases, CDC; 3 World Health Organization (WHO) Country Office, Kabul, Afghanistan; 4 WHO Country Office, Islamabad, Pakistan; 5 WHO Headquarters, Geneva, Switzerland

In 2012, the World Health Assembly declared the completion of polio eradication a programmatic emergency for global public health and called for a comprehensive polio endgame strategy (1). Afghanistan and Pakistan are two of the three remaining countries (the other is Nigeria) where circulation of indigenous wild poliovirus (WPV) has never been interrupted. This report updates previous reports (2,3) and describes polio eradication activities and progress in Afghanistan and Pakistan during January 2013–August 2014. In Afghanistan, 14 WPV cases were reported in 2013, compared with 37 cases in 2012; nine cases were reported during January–August 2014, compared with six cases during the same period in 2013. In Pakistan, 93 WPV cases were reported in 2013, compared with 58 cases in 2012; 170 cases were reported during January–August 2014, compared with 33 cases during the same period in 2013. All WPV cases reported during January 2013–August 2014 were WPV type 1 (WPV1). Vaccination campaigns have been banned since June 2012 in specific areas in Pakistan, where an estimated 300,000 children aged <5 years reside and where 69% of WPV cases have occurred in 2014. To accomplish the objectives of the Polio Eradication and Endgame Strategic Plan for 2013–2018 (1) both countries should continue to negotiate access of vaccinators to insecure and temporarily inaccessible areas, improve immunization program performance to reach more children in accessible areas, and ensure that political and health leaders at all levels are fully committed to the program, including being committed to providing financial resources needed to fully implement all the recommendations of external technical advisory groups. Both countries should also continue to strengthen cross-border collaboration to improve surveillance and case detection, coordinate outbreak response, and maximize vaccination coverage of children moving between the two countries.

## Immunization Activities

During 2013, estimated national routine vaccination coverage* of infants with 3 doses of oral poliovirus vaccine (OPV3) was 71% in Afghanistan and 72% in Pakistan (4). Routine OPV3 coverage based on parental recall and vaccination cards of children aged 6–23 months with nonpolio acute flaccid paralysis (NPAFP)† was 66% in Afghanistan (30% in the Southern, 80% in the Southeastern, and 75% in the Eastern regions), and 71% in Pakistan (25% in conflict-affected Federally Administered Tribal Areas [FATA], 37% in Balochistan, 64% in Sindh, 68% in Khyber Pakhtunkhwa [KP], and 86% in Punjab provinces).

During January 2013–August 2014, house-to-house supplementary immunization activities (SIAs)§ generally targeted children aged <5 years using different OPV formulations, including bivalent (types 1 and 3), trivalent, and monovalent (type 1) OPV. During this period, 26 SIAs were conducted in Afghanistan, including 7 national immunization days, 6 subnational immunization days, and 13 short-interval additional dose campaigns,¶ and 26 SIAs were conducted in Pakistan, including 7 national immunization days, 9 subnational immunization days, and 10 short-interval additional dose campaigns. In addition, in both Afghanistan and Pakistan, SIAs at transit posts (at border crossings between countries and borders of inaccessible districts to vaccinate children on the move), in camps, and in hosting communities targeted populations displaced by military operations in North Waziristan Agency, Pakistan, where vaccination campaigns have been banned since June 2012. In Pakistan, the number of transit posts increased from 345 in 2013 to 668 in 2014, and SIAs were conducted in Southern KP, Karachi, and Punjab to reach the displaced populations from North Waziristan. Hundreds of thousands of children aged <5 years, as well as many older children and adults, were vaccinated in both countries.

During 2013–2014, insecurity continued to hinder the ability of vaccination teams in Afghanistan and Pakistan to reach children living in temporarily inaccessible areas.** However, in SIAs conducted in 2014 in Afghanistan, the proportion of children estimated to have been missed in accessible areas (range = 3%–10%) was higher than the proportion missed because of insecurity (range = 0.2%–8.0%). In Pakistan, FATA was the area with the largest proportion of inaccessible children, with 25%–35% of children not accessible during SIAs during 2013–2014. The ability of SIAs to reach and vaccinate the targeted populations is monitored through post-SIA assessments, including lot quality assurance surveys†† (5). Improvements in SIA quality occurred in Afghanistan: lot quality assurance surveys results showed that 70%–77% of districts in 2014 passed at the ≥80% level, compared with 39%–65% of districts in 2013.

During 2013, the proportions of children aged 6–23 months with NPAFP who were “zero dose,” (i.e., had never received OPV either through routine immunization or SIAs), were 1.7% and 4.5% in Afghanistan and Pakistan, respectively, with considerable regional variation. In Afghanistan, the proportion of zero-dose children in the Southern Region declined from 14% in 2012 to 5.3% in 2013 but increased in the Eastern Region from 1% in 2012 to 5.9% in 2013; no zero-dose children were among reported NPAFP cases during 2014 to date. In Pakistan, the proportion of zero-dose children in FATA was 18% in 2012, 46% in 2013, and 61% in 2014 to date; the proportion was 1.5% in the rest of the country during 2013 and 2014.

## Poliovirus Surveillance

### Surveillance for acute flaccid paralysis (AFP)

In 2013, the annual national NPAFP rate (per 100,000 population aged <15 years) was 10.0 in Afghanistan (range among eight regions = 5.8–12.8), and 5.9 in Pakistan (range among eight provinces/regions = 1.1–12.7). The percentage of AFP cases for which adequate specimens were collected was 93% in Afghanistan (range = 83%–97%) and 89% in Pakistan (range = 82% –100%) (Table). Despite overall high AFP surveillance performance indicators,§§ genomic sequencing data indicate surveillance gaps and undetected WPV transmission in both Afghanistan and Pakistan.

What is already known on this topic?

Afghanistan and Pakistan are two of the three remaining countries (the other is Nigeria) in which indigenous wild poliovirus (WPV) transmission has never been interrupted. Conflict in both countries has made some areas inaccessible for polio eradication activities.

What is added by this report?

WPV type 1 (WPV1) transmission was highest in the Eastern Region of Afghanistan, bordering conflict-affected areas in Pakistan, including Waziristan. Although genetic sequencing data indicates that cross-border transmission from Pakistan is the biggest problem in Afghanistan, evidence indicates that undetected local transmission is occurring as well. In Pakistan, five times as many cases occurred during January–August 2013 than the same period in 2014. A total of 22 cases of circulating vaccine-derived polio virus type 2 were identified in Pakistan in 2014, signaling substantial immunity gaps. This, in addition to a high proportion of unvaccinated children in both accessible and temporarily inaccessible areas, poses an increasing risk for continued WPV transmission in Pakistan and Afghanistan.

What are the implications for public health practice?

Ongoing WPV1 transmission in Pakistan and Afghanistan poses a challenge to the achievement of global polio eradication. To achieve the objectives of the Polio Eradication and Endgame Strategic Plan for 2013–2018, both countries need to strengthen surveillance, increase cross border collaborations, use data-driven approaches to reach missed children, and evaluate the effectiveness of such approaches.

### Environmental surveillance

Environmental surveillance supplements AFP surveillance, with periodic testing of sewage samples for polioviruses. In Afghanistan, sewage sampling for polioviruses began in 2013, and WPV1 was detected for the first time in specimens from Kandahar and Nangarhar provinces in July 2014. In Pakistan, during January 2013–August 2014, a total of 551 sewage samples from 30 sampling sites in all four main provinces were tested for polioviruses. In 2014, a total of 33% (77 of 230) of sewage samples were positive for WPV1, compared with 16% (36 of 227) during the same period in 2013. WPV1 was isolated from sewage samples collected in all major cities during 2014, with the exception of Faisalabad (Punjab Province) and Islamabad. In Gadap Town, Karachi, circulating vaccine-derived poliovirus type 2 (cVDPV2) was isolated from samples collected in March and April 2014.

## WPV and cVDPV Epidemiology

**FIGURE 1:**
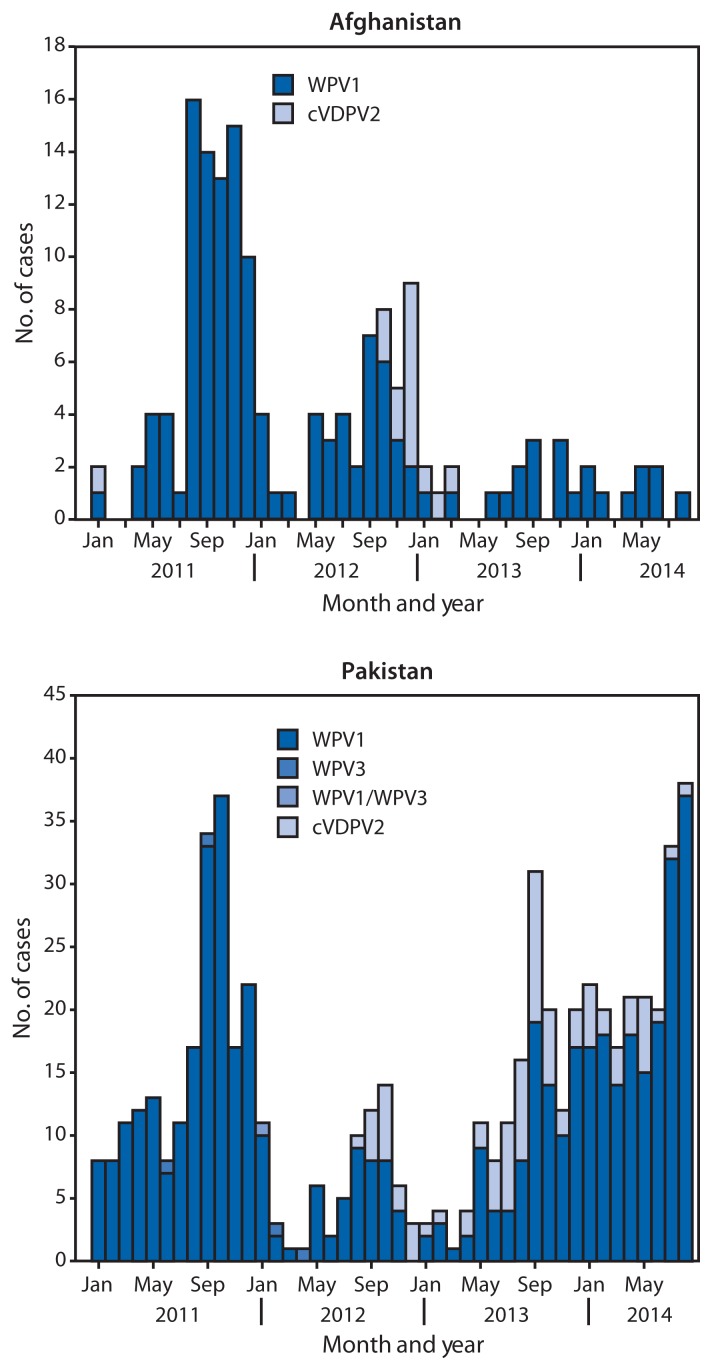
Number of cases of wild poliovirus types 1 (WPV1) and 3 (WPV3) and circulating vaccine-derived poliovirus type 2 (cVDPV2), by month — Afghanistan and Pakistan, 2011–2014

**FIGURE 2:**
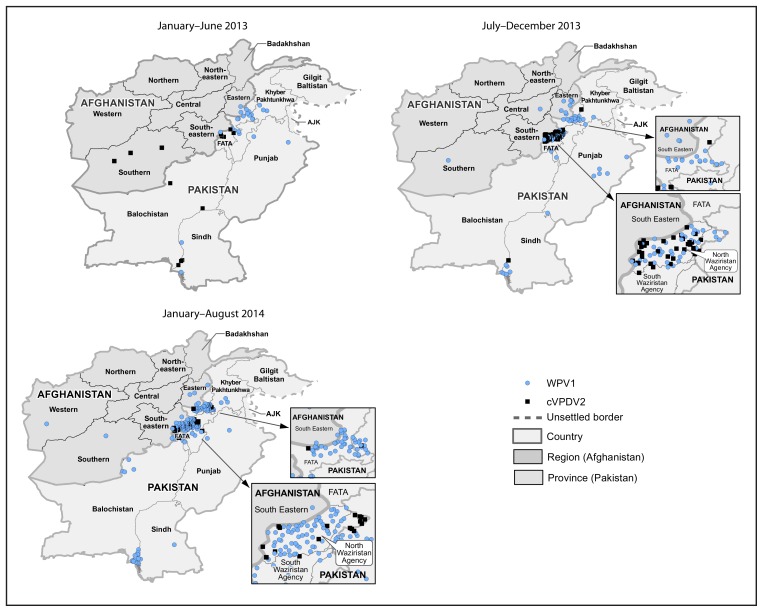
Cases of wild poliovirus type 1 (WPV1) and circulating vaccine-derived poliovirus type 2 (cVDPV2) — Afghanistan and Pakistan, January 2013–August 2014* Abbreviations: AJK = Azad Jammu and Kashmir; FATA = Federally Administered Tribal Areas. * Each dot represents one case. Dots are drawn at random within second administrative units.

In Afghanistan, 14 WPV1 cases were reported in 2013, compared with 37 cases in 2012; nine cases were reported during January–August 2014, compared with six cases during the same period in 2013 (Table, Figures 1 and 2). Of the 23 WPV1 cases reported during January 2013–August 2014, 19 (82%) were reported among children aged <36 months; of whom seven (37%) had never received OPV, three (16%) had received 1 dose, and nine (47%) received >4 doses. During this period, WPV1 cases were reported in 18 (6%) of 329 districts of Afghanistan. Of the 23 cases reported during January 2013–August 2014, a total of 17 (74%) were caused by WPV1 imported from Pakistan, and six (26%) were caused by “orphan viruses,”¶¶ indicating gaps in the quality of field AFP surveillance and missed WPV1 transmission. Two of these orphan viruses, isolated from children living in the Southern Region, belonged to the endemic Afghanistan virus previously circulating in the Southwestern Region of Afghanistan, indicating that ongoing endemic transmission had not been detected for >20 months. The other four orphan viruses originated in Pakistan. Three cVDPV2 cases were reported in 2013; the last case was reported in March 2013 (Table, Figures 1 and 2).

In Pakistan, 93 WPV1 cases were reported in 2013, compared with 58 cases in 2012; 170 cases were reported during January–August 2014, compared with 33 cases during the same period in 2013. Of the 263 WPV1 cases reported in Pakistan during January 2013–August 2014, a total of 245 (93%) were reported among children aged <36 months; 164 (67%) had never received OPV, 33 (14%) received 1–3 OPV doses, and 48 (20%) received >4 doses. In 2013, WPV1 cases were reported in 16 (10%) of 157 districts of Pakistan, compared with 27 (17%) districts in 2012, and 23 (15%) districts during January–August 2014. Of the 263 WPV1 cases reported during 2013–2014, 69% were from FATA and 16% were from KP (Table); 56% of cases in FATA were from North Waziristan Agency. During 2013–2014, a total of 70 cVDPV2 cases were reported in Pakistan (81% from North Waziristan) (Table, Figures 1 and 2); 94% were reported among children aged <36 months, of whom 68% had never received OPV. WPV type 3 has not been detected in Afghanistan or Pakistan since April 2012.

### Discussion

The number of WPV1 cases in Afghanistan decreased substantially from 2012 to 2014, after the implementation of an augmented National Emergency Action Plan in 2012 (6), and was facilitated by overall increased access to children in insecure areas. The action plan included strategies to improve program management and performance at the province and district level, including the recruitment of additional staff at district and province level, additional training of staff, and establishing a framework for holding staff accountable for their performance. The action plan also included strategies to increase access to children in insecure areas, such as the use of so-called “permanent polio teams” in the low-performing districts of the Southern Region. These teams, comprised of local staff, deliver OPV on a continuous basis, irrespective of SIAs, by making quarterly visits to all households in an assigned catchment area to increase OPV coverage. In the Southern Region, a reduction in the proportion of zero-dose children was documented, and no WPV1 cases were detected for 1 year during November 2012–October 2013. However, detection of two indigenous Afghanistan WPVs in the Southern Region in late 2013 and mid-2014, after long periods without detection, indicated ongoing undetected transmission and weaknesses in AFP surveillance. A ban on immunizations in Helmand Province (Southern Region), imposed by local authorities during March–August 2014, was lifted during the last week of August, and a series of SIAs have been conducted in Helmand since then. Undetected local transmission demonstrated by genetic sequence data, ongoing poliovirus transmission in bordering areas of Pakistan, and cross-border movement from Pakistan, place Afghanistan at risk for continued WPV transmission. In addition, access to children during SIAs in certain areas of the Eastern Region, particularly in Kunar Province, remains challenging. Despite these challenges, major improvements were made in vaccination coverage in 2014. Strengthening surveillance in the Southern Region and increasing vaccination coverage in all regions is critical to achieve the goal of interrupting all poliovirus transmission in Afghanistan.

In Pakistan, a five-fold increase in the number of reported WPV1 cases occurred in 2014, compared with the same period in 2013; 87% of cases in 2014 were reported in FATA and KP. The increase in the number of cities with WPV1 and cVDPV2 isolated from sewage samples indicates widespread exportation of poliovirus from FATA and KP; however, increased vaccination coverage through numerous SIAs has prevented outbreaks or sustained transmission elsewhere in Pakistan. To achieve polio eradication, new ways of reaching children living in temporarily inaccessible areas in Pakistan are needed, including negotiating ways to ensure the safety of vaccination teams within conflict-affected areas and continued use of transit vaccination teams at borders of inaccessible areas. In addition, full government ownership of the polio eradication program at all administrative levels in Pakistan is crucial.

Interruption of all circulation of indigenous WPV in Afghanistan is within reach. However, the situation in Pakistan is threatening the global polio eradication efforts. Given the current situation in Pakistan, it is highly unlikely that all necessary programmatic steps can be taken to interrupt WPV transmission during 2014. However, if the government of Pakistan implements all the recommendations provided by the polio technical advisory group and manages the eradication program more effectively, the low season of the last quarter of 2014 and the first quarter of 2015 provides an opportunity to further accelerate activities to limit or stop all WPV transmission. Unless Pakistan makes substantial improvements to its program and controls WPV spread within its borders, the global efforts to eradicate polio will be undermined.

## Figures and Tables

**TABLE:** Acute flaccid paralysis (AFP) surveillance indicators and reported cases of wild poliovirus (WPV) and circulating vaccine-derived poliovirus (cVDPV), by region, period, and poliovirus type — Afghanistan and Pakistan, January 2013–August 2014

	AFP surveillance indicators (2013)			Reported WPV1 cases*				Reported cVDPV2 cases*		
			
Country/Area	No. of AFP cases	Nonpolio AFP rate†	% with adequate specimens§	Jan–Jun 2013	Jul–Dec 2013	Jan–Aug 2014	Total WPV1	Jan–June 2013	July–Dec 2013	Jan–Aug 2014
**Afghanistan**	**1,897**	**10.0**	**93**	**3**	**11**	**9**	**23**	**0**	**0**	**0**
Badakhshan	62	11.3	96	0	0	0	**0**	0	0	0
Northeastern	252	11.7	96	0	0	0	**0**	0	0	0
Northern	279	11.9	92	0	0	0	**0**	0	0	0
Central	346	8.0	96	0	1	0	**1**	0	0	0
Eastern	176	9.3	93	3	9	5	**17**	0	0	0
Southeastern	119	5.8	94	0	0	2	**2**	0	0	0
Southern	337	10.2	83	0	1	1	**2**	3	0	0
Western	326	12.8	97	0	0	1	**1**	0	0	0
**Pakistan**	**4,658**	**5.8**	**89**	**21**	**72**	**170**	**263**	**10**	**38**	**22**
Azad Jammu Kashmir	50	3.1	94	0	0	0	**0**	0	0	0
Gilgit-Baltistan	8	1.1	88	0	0	0	**0**	0	0	0
Islamabad	17	2.7	100	0	0	0	**0**	0	0	0
Khyber Pakhtunkhwa	875	8.2	84	5	6	31	**42**	0	0	3
Punjab	2,257	5.4	92	2	5	2	**9**	0	0	0
Balochistan	195	5.1	86	0	0	4	**4**	2	0	0
Sindh	955	5.3	91	2	8	16	**26**	3	1	0
Federally Administered Tribal Areas	301	12.7	82	12	53	117	**182**	5	37	19

* Data as of October 17, 2014.

† Per 100,000 children aged <15 years.

§ Two specimens collected ≥24 hours apart, both within 14 days of paralysis onset, and shipped on dry ice or frozen packs to a World Health Organization–accredited laboratory, arriving in good condition (without leakage or desiccation).

## Footnotes

* Includes vaccines given through the Expanded Program of Immunization only and not those given through supplemental immunization activities.

† Vaccination histories of children aged 6–23 months with acute flaccid paralysis who do not test WPV-positive are used to estimate OPV coverage of the overall target population and to corroborate national reported routine immunization coverage estimates.

§ Mass campaigns conducted for a brief period (days to weeks) in which 1 dose of OPV is administered to all children aged <5 years, regardless of vaccination history. Campaigns can be conducted nationally or in sections of the country.

¶ Short-interval additional dose campaigns are used for case-response vaccination after WPV cases, or during negotiated periods of nonviolence in otherwise inaccessible areas, to vaccinate children with a monovalent or bivalent OPV dose, which is administered within 1–2 weeks of the previous dose.

** Temporarily inaccessible areas are those where vaccination teams are temporarily unable to operate because of security concerns or bans on vaccination.

†† Lot quality assurance sampling is a rapid survey method used to assess the quality of vaccination coverage after SIAs in predefined areas, such as a health districts (known as “lots”), using a small sample size. The lot quality assurance sampling method involves dividing the population into “lots” and randomly selecting persons in each lot. If the number of unvaccinated persons in the sample exceeds a preset decision value, then the lot is classified as having an unsatisfactory level of vaccination coverage, and mop-up activities are recommended. If ≥80% of lots meet the cutoff, the area/district is classified as having “passed.”

§§ The quality of AFP surveillance is monitored by performance indicators that include 1) detection rate of NPAFP cases and 2) proportion of AFP cases with adequate stool specimens. World Health Organization (WHO) operational targets for countries with endemic poliovirus transmission are an NPAFP detection rate of at least two cases per 100,000 population aged <15 years and adequate stool specimen collection from >80% of AFP cases, where adequate specimens are two specimens collected ≥24 hours apart, both within 14 days of paralysis onset, and shipped on dry ice or frozen packs to a WHO-accredited laboratory, arriving in good condition (without leakage or desiccation).

¶¶ Any wild-type poliovirus that is >1% divergent in the VP1 region from the most closely related isolate is defined as an “orphan” poliovirus and considered indicative of low AFP surveillance sensitivity.
